# Kleptomania

**Published:** 1885-04

**Authors:** 


### KLEPTOMANIA.

Sergeant Ballantyne, a leading English lawyer, cites in his memoirs the following case of this disease: the wife of an eminent physician was suspected of stealing from a draper's shop. She was watched, followed and searched, and several small articles found in her dress. She was given into custody, and went through the ordeal of an inquiry at the police court and was committed for trial. The presiding judge was thoroughly impartial, knew all the law necessary for his position, but it was not very well packed in his brain, and the particles constantly came out at wrong times and places. The case, however, could hardly have been confused; the facts were perfectly clear; the whole of the lady's life, as far as its history was known, was not only free from reproach, but thoroughly rational. The only point that could be relied upon for the defense was, that the articles stolen were so trivial that no sane object could exist for intentional theft, and the only suggestion that could be made in her favor was that she was not responsible for her actions, being compelled by an uncontrollable impulse, that she was the victim of kleptomania—not a very popular defense before a jury of tradesmen. However, after having been locked up for some hours, they were ultimately discharged without giving a verdict, a result arising probably more from compasslon for the lady's husband than any doubts about the facts. Sergeant Ballantyne says that he thought at the time that if, either to herself or to the doctor, it would have been the kinder couse, and subsequent circumstances showed that in reality her conduct was attributable to insane influences, although she knew thoroughly well that she was acting wrongly. She died very shortly after the ordeal she had undergone, broken down in health and spirit, with the shame and disgrace, and the sergeant was consulted, after her death had taken place, by Dr. R., under the following circumstances: Every drawer and cupboard in the house was found to be full of new goods, which she must have been in the habit of abstracting during many years, and in every instance they were contained in their original wrappers. Mrs. R. was a religious woman, and every Sunday she listened with respect and veneration to the lessons taught in the church, and fully realized the commandment of "Thou shalt not steal." And it is clear that she, by the acts that she committed, incurred danger and obtained no advantage. The sergeant advised Dr. R. not to make the discovery public, and the articles found were distributed amongst different charitable institutions. Can any one doubt that insanity irresistibly controlled her conduct? Now, while it cannot be denied that women (theoretically respectable), while shopping, often pilfer small articles of value, to them their conduct is very different from that of the lady described in the citation.

